# Confounding Effect of Hepatic Carboxylesterase 1 (CES1) Variability on Clopidogrel Oxidation

**DOI:** 10.1021/acs.molpharmaceut.5c00462

**Published:** 2025-11-13

**Authors:** Sandhya Subash, Dilip K. Singh, S. Cyrus Khojasteh, Bernard P. Murray, Michael A. Zientek, Robert S. Jones, Priyanka Kulkarni, Bill J. Smith, Bhagwat Prasad

**Affiliations:** † College of Pharmacy and Pharmaceutical Sciences, Washington State University (WSU), Spokane, Washington 99202, United States; ‡ Drug Metabolism and Pharmacokinetics, Genentech, Inc., South San Francisco, California 94080, United States; § Drug Metabolism, Gilead Sciences, Inc., Foster City, California 94404, United States; ∥ Drug Metabolism and Pharmacokinetics, Takeda Development Center Americas, Inc., San Diego, California 92121, United States; ⊥ Drug Metabolism and Pharmacokinetics, Takeda Pharmaceuticals, Inc., Cambridge, Massachusetts 02139, United States; # Terminal Phase Consulting LLC, Colorado Springs, Colorado 94404, United States

**Keywords:** CES1, CYP, clopidogrel, fraction metabolized

## Abstract

Clopidogrel, a frequently used prodrug, is converted to its active metabolite through the intermediate 2-oxo-clopidogrel by cytochrome P450 (CYP) enzymes, which accounts for only 5%–15% of its metabolism. Majority of the clopidogrel dose (85%–90%) is extensively hydrolyzed to its inactive metabolite, clopidogrel carboxylic acid by carboxylesterase 1 (CES1). In vitro studies suggest the involvement of multiple CYP isoforms, with CYP1A2, CYP2C19, and CYP2B6 identified as major contributors to 2-oxo-clopidogrel formation. While CYP2C19 genetic polymorphisms are often highlighted as the primary factor contributing to variability in the clopidogrel response, the confounding role of CES1 variability on clopidogrel oxidation is less well understood. Our study utilizing proteomics-informed scaling highlights the importance of accurate estimation of the fraction metabolized (*f*_m_) by CES1 and CYPs in clopidogrel metabolism. The results also indicate that differential subcellular localization of these enzymes and technical variability in sample preparation can influence *f*_m_ estimation, suggesting that HLM may not be an ideal model for investigating dual substrates of CYPs and CES. Quantitative proteomics and activity assays revealed significant variability in the absolute content and activities of CES1 and CYP enzymes across HLM donors (*n* = 10), which affected the estimation of *f*_mCES_ versus *f*_mCYP_. Human hepatocyte assay, which represents a CYP versus CES abundance ratio similar to that in liver tissue, demonstrated the critical roles of CYP3A4 and CES1 abundance in the 2-oxo-clopidogrel formation rate. Further, enzyme kinetic studies identified CYP3A4 as the primary contributor to 2-oxo-clopidogrel formation, but multiple other enzymes, including CYP2C9, were identified as contributors. Overall, our findings emphasize the need for accounting for variability in both CES1 and CYP enzymes to improve *f*_m_ estimation in the in vitro to in vivo extrapolation of dual substrates of CYP/CES such as clopidogrel.

## Graphical abstract

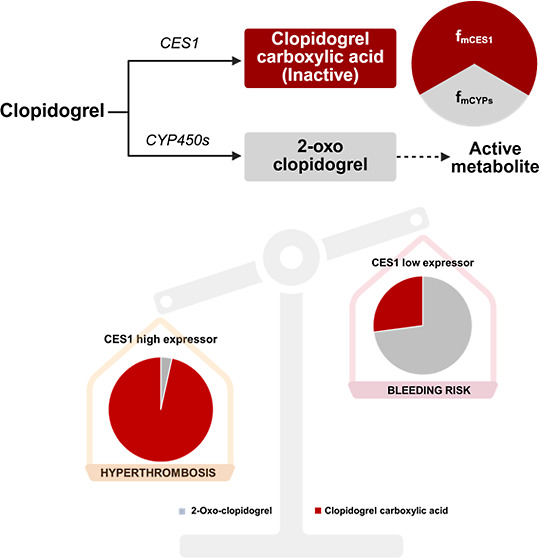

## 1 Introduction

Clopidogrel (Plavix) is a widely prescribed antiplatelet drug used for the management of cardiovascular diseases, including unstable angina, atherothrombosis, and myocardial infarction.^1^ Administered as an inactive prodrug, clopidogrel requires a 2-step bioactivation to an active metabolite that contains a reactive thiol group (Figure 1). The active metabolite irreversibly binds to the P2Y12 receptor via the formation of a disulfide bond inhibiting platelet aggregation.^2,3^ Following oral administration, approximately 50% of the drug is absorbed and undergoes extensive first-pass metabolism in the liver.^4^ The metabolism of clopidogrel is a complex process that involves several enzymes (Figure 1). Around 85%–90% of clopidogrel fails to be activated because it is hydrolyzed by liver carboxylesterase 1 (CES1) to the inactive metabolite clopidogrel carboxylic acid (CCA).^5^ CCA is converted to its acylglucuronide metabolite mainly by the enzyme UGT2B7, with additional contributions from UGT2B4 and UGT2B17.^6^ A small proportion (5%–15%) of clopidogrel undergoes sequential oxidative metabolism. For the first step, which involves the formation of 2-oxo-clopidogrel, multiple cytochrome P540 (CYP) isoforms, such as CYP1A2, CYP2B6, and CYP2C19, have been reported to be involved.^7,8^ Subsequently, the active metabolite is generated via oxidation of the thiolactone moiety to a sulfenic acid intermediate, followed by a two-step reduction by glutathione.^9–11^ In addition to CYP2B6 and CYP2C19, that are reported to be involved in the first step, two other CYPs mainly, CYP2C9 and CYP3A4, have been shown to contribute to the formation of the active metabolite.^8^

**Figure 1:**
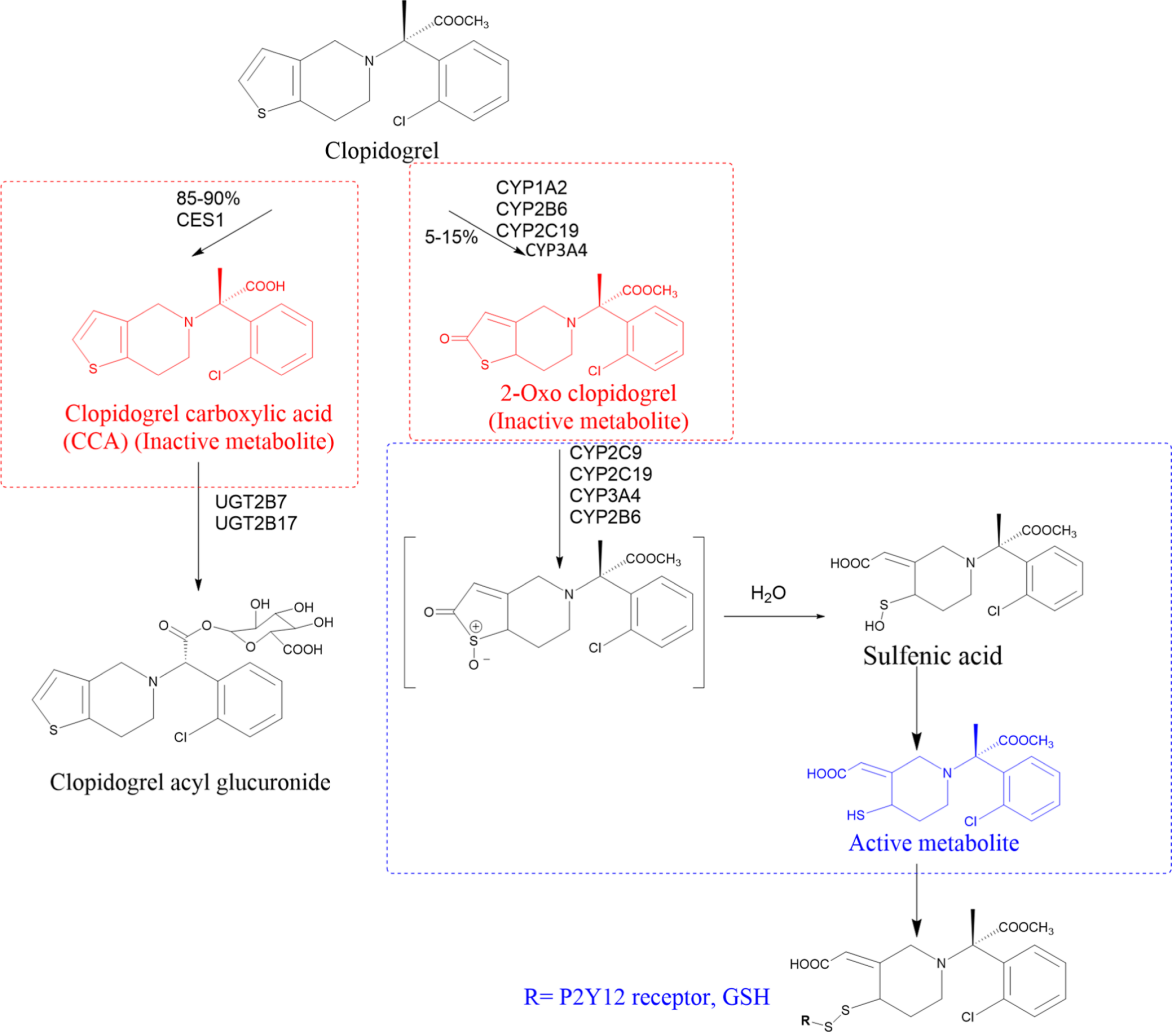
Metabolic pathways of clopidogrel.

While in general, clopidogrel has been proven to be safe and effective, it exhibits significant interindividual variability in its pharmacokinetics and pharmacodynamics.^12^ A lack of efficacy, also known as “clopidogrel resistance”, has been reported to affect 5%–44% of patients receiving the standard dose of 75 mg, whereas some patients also experience drug-induced bleeding due to excessive inhibition of platelets.^13,14^ The interindividual variability has been associated with covariates such as age, body weight, and sex but more importantly to genetic polymorphisms in CYP enzymes, particularly the CYP2C19 variants.^15–17^ In Hawaii, a significant proportion of patients suffering from acute myocardial infarction and on clopidogrel therapy experienced a higher incidence of mortality, where the standard 75 mg dose of clopidogrel was found to have variable efficacy in native Hawaiians due to the presence of CYP2C19 loss-of-function alleles in this population.^18^ A meta-analysis evaluating clopidogrel resistance in patients across diverse population suggests an association between CYP2C19*2 gene polymorphism and clopidogrel resistance in Asian population.^19^ Extensive in vitro and in vivo studies linking CYP2C19 genotype to clopidogrel response variability have led the Clinical Pharmacogenetics Implementation Consortium (CPIC) to recommend genotype-guided clopidogrel therapy based on the CYP2C19 status of the patient.^20,21^

Despite the large body of evidence implicating CYP2C19 genetic polymorphisms in variability to clopidogrel response, there are limited investigations on the potential role of genetic variability or inhibition of CES1.^22,23^ A review on factors affecting clinical pharmacokinetics (PK) and pharmacodynamics (PD) of clopidogrel highlights the need for more research on the impact of CES1 variability on clopidogrel response.^24^ A knowledge gap exists regarding the possible metabolic switching in cases of variability in CES1, which may lead to toxicity from higher levels of the active metabolite in individuals with nonfunctional CES1 alleles, whereas reduced efficacy may be observed when the ratio of CES to CYP is higher due to increased CCA formation. With respect to dual substrates of CYP and CES enzymes, in vitro models such as microsomes and cytosol may not account for the differential subcellular localization for translation to an in vivo system. Therefore, it is important to evaluate the utility of routinely used in vitro systems such as liver fractions and hepatocytes alone or in combination for determining the fractional contributions of CES and CYP enzymes for dual enzyme substrates.

To address these knowledge gaps, we aimed to evaluate the relative contributions of the enzymes involved in the metabolism of clopidogrel, a dual substrate of CYP and CES. We employed proteomics to determine the content of the various CYP isoforms and CES1 in human liver microsomes (HLM), human hepatocytes, and recombinant enzyme preparations. Further, activity assays were performed to estimate the relative formation of CCA and 2-oxo-clopidogrel and the *f*_mCYP_ toward the formation of 2-oxo-clopidogrel. Additionally, we looked at the applicability of HLM as an in vitro model, given the differential localization of CYP and CES enzymes.

## 2 Materials and Methods

### 2.1 Chemicals and Reagents

Methanol, dimethyl sulfoxide (DMSO), mass spectrometry (MS)-grade acetonitrile (ACN), potassium dihydrogen phosphate, dipotassium hydrogen phosphate, and formic acid were procured from Thermo Fisher Scientific (Fair Lawn, NJ). Acetone was purchased from Sigma-Aldrich (St Louis, MO). The bicinchoninic acid (BCA) assay kit for total protein quantification was purchased from Pierce Biotechnology (Rockford, IL). Ammonium bicarbonate (purity, 98%), dithiothreitol (DTT), iodoacetamide (IAA), and MS-grade trypsin were also procured from Thermo Fisher Scientific (Rockford, IL). Bovine serum albumin (BSA) was purchased from Calbiochem (Billerica, MA). Analytical standard grade clopidogrel hydrochloride (≥98%) was procured from Cayman Chemicals (Ann Arbor, MI), while diclofenac was procured from Sigma-Aldrich (St Louis, MO). Analytical standards of CCA and 2-oxo-clopidogrel were purchased from Toronto Research Chemicals (ON, Canada). HLM from individual donors (*n* = 10) were procured from BioIVT (Baltimore, MD). Pooled HLM (mixed sex, *n* = 150), human liver S9 fractions (HLS9, *n* = 50), recombinant EasyCYPs with high reductase +b5 (rCYP1A2, rCYP2B6, rCYP2C9, rCYP2C19, and rCYP3A4), and CES1 (rCES1) were procured from Xenotech LLC (Kansas, MO). Cryopreserved adult hepatocytes (*n* = 3 donors), hepatocyte thawing media (INVITROGRO HT), and incubation media (INVITROGRO KHB) were gifted by BioIVT (Baltimore, MD).

### 2.2 Protein Extraction and Digestion of Samples for CYP and CES Quantification

The total protein concentration in individual and pooled HLM, pooled human liver S9 (HLS9) fractions, rCYPs, and rCES1 was measured using a BCA assay kit. The cryopreserved adult hepatocytes (*n* = 3; 2 males and 1 female) were thawed using the manufacturer protocol, and the cells were counted and diluted using INVITROGRO KHB media. One million hepatocytes were mixed with 300 μL of solubilization buffer of the Mem-PER Plus Membrane Protein Extraction Kit and incubated for 60 min at 300 rpm (4 °C). Approximately 80 μL of liver fraction or recombinant preparations (containing 80 μg of protein) and hepatocyte sample (containing 1 million hepatocytes) were then digested according to the protocol reported elsewhere.^25^ To each 80 μL sample were added 100 mM ammonium bicarbonate (30 μL), 2 μg/mL BSA (20 μL), and 50 mM DTT (10 μL) followed by gentle shaking at 300 rpm for 10 min at 95 °C for denaturation and reduction. The samples were cooled to room temperature for 10 min and then alkylated with 10 μL of 500 mM IAA for 30 min in the dark. Protein precipitation was performed by adding 1 mL of ice-cold acetone and incubating at −80 °C, followed by centrifugation at 16,000*g* and 4 °C for 15 min. The pellet was washed with ice-cold methanol and centrifuged again under the same condition. The resulting pellet was dried at room temperature and resuspended in 60 μL of 50 mM ammonium bicarbonate. Trypsin digestion was carried out by adding 20 μL of trypsin and incubating it for 16 h at 37 °C with gentle shaking (300 rpm) using an Eppendorf Thermo mixer. The digested sample was quenched with 5 μL of 0.5% formic acid, vortex-mixed, and centrifuged at 16,000*g* and 4 °C for 10 min, and the supernatant was transferred into a liquid chromatography mass spectrometry (LC-MS) vial for analysis.

### 2.3 Quantitative Proteomic Analysis of Digested Samples Using Total Protein Approach

An optimized LC-MS/MS method was used to quantify CYP1A2, CYP2B6, CYP2C8, CYP2C9, CYP2C19, CYP3A4, and CES1. Global proteomics data acquisition of the digested samples was performed using an EASY-nLC 1200 series system coupled with a Q-Exactive-HF MS instrument (Thermo Scientific, San Jose, CA) in data-independent acquisition (DIA) and positive ionization modes. The digested sample 1 μL (1 μg protein) was injected, and the peptides were separated using a PepMap RSLC C18 column (2 μm, 250 × 0.075 mm, Thermo Scientific). The column was maintained at 40 °C, with a flow rate of 300 nL/min, using 0.1% formic acid in water and 0.1% formic acid in 80% acetonitrile as mobile phases A and B, respectively. The gradient program (%B) is as follows: 0–5 min (2%–6%), 5–60 min (6%–30%), 60–65 min (30%–100%), and 65–80 min (100%). The EASY-Spray source was operated at a spray voltage of 1.7 kV. The MS was operated in the full scan mode with a resolution of 120,000 for MS1 and 30,000 for DIA, an automatic gain control target of 3 × 10^6^ for MS1 and 1 × 10^6^ for DIA, a maximum injection time of 55 ms, a capillary temperature of 275 °C, and a 50 S-lens RF level. The *m*/*z* scan range was set to 350–1100.

The MS data (RAW files) were analyzed using DIA-NN software (v1.8.1) (https://www.nature.com/articles/s41592-019-0638-x) with the *Homo sapiens* proteome library for human hepatocyte samples. The maximum number of missed trypsin cleavages was set to two with a maximum of five variable modifications and a false discovery rate of 1%. The following modifications were included: carbamidomethylation of cysteine residues and acetylation of protein N-termini as fixed modifications, with oxidation of methionine as a variable modification. All other parameters were set to the default DIA-NN settings.

Protein abundance was determined using the total protein approach (TPA).^26,27^ The protein concentration (pmol/mg membrane protein) was calculated using the spectral peptide intensities (raw intensities) and protein molecular weight (eq 1).

1 $${\left[Protein\right]}_{i}=\frac{{MS\;intensity}_{i}}{total\;MS\;intensity\times {MW}_{i}}$$

where MS intensity_*i*_ and total MS intensity refer to the sum of MS spectral intensities of all unique (proteotypic) peptides of protein i and the sum of MS spectral intensities of all the peptides in the sample, respectively, and MW_i_ is the molecular weight of protein i.

### 2.4 2-Oxo-clopidogrel and Clopidogrel Carboxylic Acid Formation Assay in Pooled and Individual Liver Subcellular Fractions

CYP-mediated 2-oxo-clopidogrel and CES-mediated CCA formation activities were determined in pooled HLM and HLS9 fractions in 0.1 M potassium phosphate buffer, pH 7.4. The protein concentration was maintained at 0.5 mg/mL, and all incubations were conducted in triplicates in a water bath shaker at 37 °C with incubation volumes of 100 μL each. The incubations were fortified with NADPH (1 mM final concentration), and the metabolite formation reactions were initiated by adding clopidogrel at reported *V*_max_ concentration for both CES1 and CYP pathways (i.e., 150 μM).^5,8^ Reactions were quenched at various time points (5, 10, 15, 30, 45, and 60 min) by adding 200 μL of acetonitrile containing diclofenac as an internal standard (33.3 nM, final concentration). Samples were centrifuged at 10,000*g* for 5 min (4 °C), and the supernatants were collected and diluted 10-fold using a 75:25 (0.1% formic acid in ACN/0.1% formic acid in water) solution before being transferred to an LC-MS vial for analysis. Calibration standards were prepared by adding stock solutions (1–2000 μM in 25% DMSO) of 2-oxo-clopidogrel and CCA in buffer to achieve final concentrations of 1–1000 nM processed in the same manner as the incubation samples.

Incubations in individual donor HLM samples were conducted according to the assay protocol described above. Reactions were terminated at 30 and 60 min by adding 200 μL of acetonitrile containing the internal standard, and samples were processed as described above.

### 2.5 Formation of 2-Oxo-clopidogrel and CCA in Human Hepatocytes from Individual Donors

The formation of 2-oxo-clopidogrel and CCA was evaluated in cryopreserved human hepatocytes from three individual donors. Hepatocytes in suspension (0.3 × 10^6^ viable cells) were incubated with substrate concentrations approximating the *K*_m_ values determined in our study for both CES1 and CYP pathways, using clopidogrel (15 μM) in a total incubation volume of 300 μL. The incubations were carried out for 30 min in a 5% CO_2_ incubator at 37 °C. Reactions were terminated at the end of 30 min, and samples were processed as described above. The samples were analyzed by a nanoflow LC coupled to a Thermo Q-Exactive-HF high-resolution mass spectrometer (HRMS) using parallel reaction monitoring (PRM) scan mode. The mass spectrometer was equipped with a standard EASY-Spray ion source, and a PepMap RSLC C18 column (2 μm, 250 mm × 0.075 mm, Thermo Scientific) was used as described previously. LC conditions were set at 300 nL/min flow rate and 1 μL injection volume using mobile phase (A): 0.1% formic acid in water and (B): 0.1% formic acid in 80% acetonitrile. The PRM-MS parameters are given (Supplementary Table 1). The area ratios of 2-oxo-clopidogrel to the internal standard and of CCA to the internal standard were determined for each sample.

### 2.6 Enzyme Kinetics of 2-Oxo-clopidogrel and CCA Formation in rCYPs and rCES Preparations

For 2-oxo-clopidogrel formation, a 5 min incubation time was selected based on the results of the above experiment with pooled HLM, which indicated it was within the linear range for metabolite formation. HLM and rCYP were incubated at 0.2 mg/mL with a range of clopidogrel concentrations (0.3–160 μM) at 37 °C for 5 min, with the final concentration of each rCYP in the incubation mix set to 20 pmol. For CCA formation, HLM and rCES1 were incubated at a final concentration of 0.1 mg/mL with a range of concentrations of clopidogrel (0.9–240 μM) at 37 °C for 30 min. In each case, at the end of the incubation time, the reaction was stopped, and the samples were processed using the 2-oxo-clopidogrel and CCA formation activity protocol described above.

### 2.7 Inhibition of 2-Oxo-clopidogrel Formation Using Sulfaphenazole and CYP3cide

The formation of 2-oxo-clopidogrel was assessed in the presence and absence of CYP2C9 and CYP3A4 inhibitors, sulfaphenazole, and CYP3cide, respectively, using clopidogrel at concentrations approximately nearing the *K*_m_ for each enzyme, i.e., 20 μM. The incubations were carried out using pooled HLM in 0.1 M potassium phosphate buffer pH 7.4 at a final protein concentration of 0.2 mg/mL. All incubations were conducted in triplicates in a water bath shaker at 37 °C with incubation volumes of 100 μL each. For evaluating CYP2C9 inhibition, HLM was preincubated with sulfaphenazole (10 μM) for 5 min, prior to initiation of the reaction with addition of NADPH (1 mM) and clopidogrel (20 μM). In the case of CYP3cide (2 μM), a 30 min preincubation was carried out in the presence of NADPH (1 mM), prior to initiation of the reaction with the addition of clopidogrel (20 μM). Additionally, a parallel set of control incubations were performed in the absence of inhibitors and in the presence of solvent (0.1% DMSO). In each case, the reactions were terminated at the end of 5 min, and the samples were processed as described above. The area ratios of 2-oxo-clopidogrel or CCA to the internal standard were determined for each sample. The percentage inhibition was calculated by comparing the ratios in the respective inhibitor-treated group to that in the solvent-treated group.

### 2.8 LC-MS/MS Analysis of 2-Oxo-clopidogrel and CCA Formation in HLM, HLS9, rCYPs, and rCES

The samples were analyzed using an LC-MS/MS system consisting of microflow LC and Xevo-TQ-XS MS systems (Waters, Milford, MA). An Acquity UPLC HSS T3 C18 column (1.8 mm, 1 mm × 100 × 100 mm) equipped with a guard column (Vanguard precolumn, 1.8 mm, 2.1 × 5 mm) was used for the analysis. Fifty microliters per minute flow rate of mobile phases A (water containing 0.1% formic acid) and B (acetonitrile containing 0.1% formic acid) were run using the following gradient program: 0–0.5 min (5%–25% B), 0.5–6.5 min (25%–60% B), 6.5–7.5 min (60%–90% B), 7.5–10 min (90% B), and 10–13 min (95%–5% B). The mass spectrometer was operated in multiple reaction monitoring (MRM) and positive ionization modes with a cone voltage of 30 V using an electrospray ionization source. The MRM transitions were 2-oxo-clopidogrel (*m*/*z* 338.0 → 155.0 and *m*/*z* 338.0 → 183.0; collision energy [CE], 25 eV), CCA (*m*/*z* 308.0 → 169.0, *m*/*z* 308.0 → 198.0; and *m*/*z* 308.0 → 152.0; CE, 20 eV), and diclofenac (*m*/*z* 296.0 → 214.0; CE, 25 eV).

### 2.9 Data Analysis

The relative expression factor (REF) for the rCYPs and rCES was calculated by using eq 2.

2 $$REF=\frac{CYP\;\text{or}\;CES\;content\;in\;pooled\;HLM\;\left(per\;mg\;protein\right)}{CYP\;\text{or}\;\mathrm{CES content in the recombinant system (per mg protein)}}$$

The enzyme kinetic parameters (Michaelis–Menten constant, *K*_m_, and maximum rate of the reaction, *V*_max_) for 2-oxo-clopidogrel and CCA formation were determined by fitting the Michaelis–Menten equation (eq 3) to the data using GraphPad Prism (ver. 8.4.3) (La Jolla, CA), where *S* is the total substrate concentration.

3 $$Rate\;of\;metabolism=\frac{V_{\max}\times S}{K_{m}+S}$$

Assuming *S* ≪ *K*_m_, *V*_max_/*K*_m_ for 2-oxo-clopidogrel formation was scaled to CL_int_ (μL/mg microsomal protein/min) by multiplying with the content (pmol/mg microsomal protein) of the respective CYPs in pooled HLM. The CL_int, total_ for each isoform was then multiplied with the respective REF values, and fraction metabolized (*f*_m_) is calculated as shown in eq 4.

4 $$f_{m,{CYP}_{i}}=\frac{Scaled\;{CL}_{\mathrm{int},\;total,\;2-oxo-clopidogrel\;{CYP}_{i}}}{Scaled\;activity\;{CYP}_{\text{all}}}\times 100$$

### 2.10 Statistical Analysis

Linear regression analyses were performed, and their corresponding p values were determined using Excel. The standard errors of the coefficients were obtained from the regression output, and two-tailed *p*-values were derived from the t-statistics for each coefficient. A p-value < 0.05 was considered statistically significant. The rates of formation of 2-oxo-clopidogrel and CCA in individual human liver microsomes at 30 and 60 min were compared using the Wilcoxon signed-rank test in GraphPad Prism (version 8.4.3, La Jolla, CA).

## 3 Results

### 3.1 Protein Content of CYP and CES Enzymes in Recombinant Human Systems and the Liver Subcellular Fractions

The protein contents of CYP1A2, CYP2B6, CYP2C9, CYP2C19, CYP3A4, and CES1 determined by quantitative global proteomics are shown (Figure 2a–f). The content of most CYPs and CES1 (expressed as pmol/mg total protein) varied considerably across HLM samples from individual donors (*n* = 10). In the case of CYP2C9, the abundances were found to be consistent across donors (Figure 2c). While the proteomics data for these were obtained in duplicate, the replicates were reproducible (Supporting Information Figure S1a,b). In the individual HLM samples, the content of CES1 showed a poor correlation (*r*2 = 0.0003 and *p* value = 0.96) with the endoplasmic reticulum (ER) membrane marker protein (calnexin) and a statistically significant correlation (*r*2 = 0.43 and *p* < 0.05) with the ER luminal marker protein (calreticulin) (Supporting Information Figure S2a,b). The ratios of calreticulin to calnexin in the individual HLM samples showed around three-fold variability (Supporting Information Figure S2c), indicating differential ER luminal to membrane compositions due to variable release of luminal contents during the process of preparation of HLM across donors.

**Figure 2:**
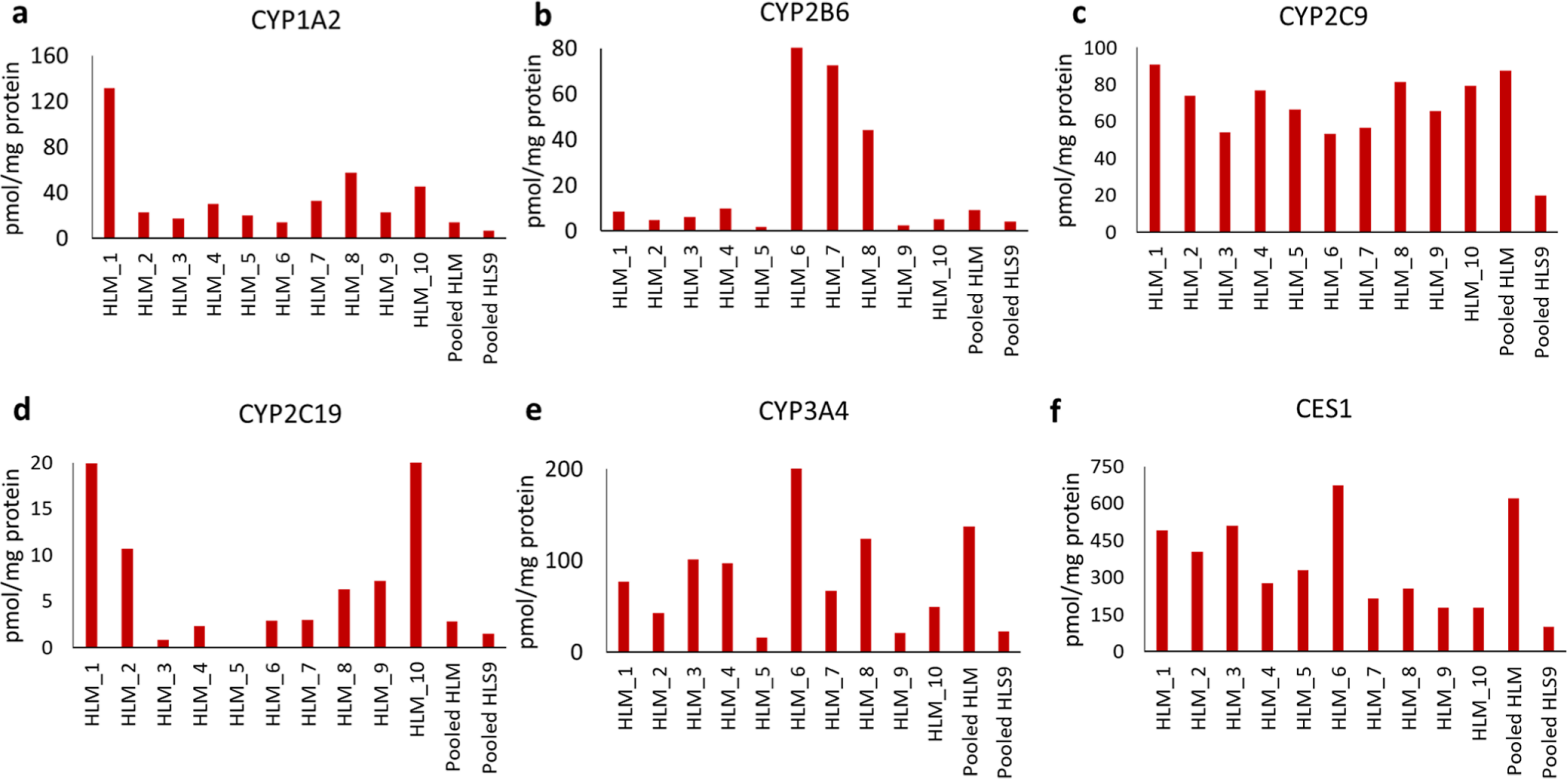
Relative abundances of CYP1A2, CYP2B6, CYP2C9, CYP2C19, and CYP3A4 and CES1 in individual HLM (*n* = 10), pooled HLM (*n* = 150) and pooled HLS9 (*n* = 50) using TPA-based global proteomics.

In the recombinant systems, the CYP and CES contents were 5- to 500-fold higher than those in HLM, with REF values ranging from 0.002 to 0.2 (Table 1).

**Table 1:** Relative Abundances of CYP1A2, CYP2B6, CYP2C9, CYP2C19, CYP3A4, and CES1 in the Recombinant Human Systems and the Corresponding REF Values

rCYP/CES isoform	Abundance (nmol/mg protein)	REF
CYP1A2	6.13	0.0028
CYP2B6	1.7	0.0054
CYP2C9	4.09	0.0214
CYP2C19	1.55	0.0018
CYP3A4	3.89	0.0353
CES1	2.69	0.175

### 3.2 Formation of 2-Oxo-clopidogrel and CCA in Pooled and Individual Liver Subcellular Fractions

The rate of formation of 2-oxo-clopidogrel and CCA in HLM (pmol/min/mg protein) was two-fold higher than in HLS9 (Figure 3a,b). However, upon normalizing the CCA formation rate with the absolute CES1 content, the metabolite formation rate (pmol/min/pmol CES1) in HLS9 was two-fold higher than that in HLM (Figure 3c). Using HLM from individual donors, the formation rates of 2-oxo-clopidogrel (pmol/min/mg protein) were significantly higher (*P* < 0.05) in 30 min incubations compared to 60 min incubations (Figure 3d). In contrast, the CCA formation rates were higher at 60 min than at 30 min (Figure 3e). A significant correlation (*P* < 0.05) was observed between CES1 content and the rate of CCA formation in individual HLM samples (Figure 3f).

**Figure 3:**
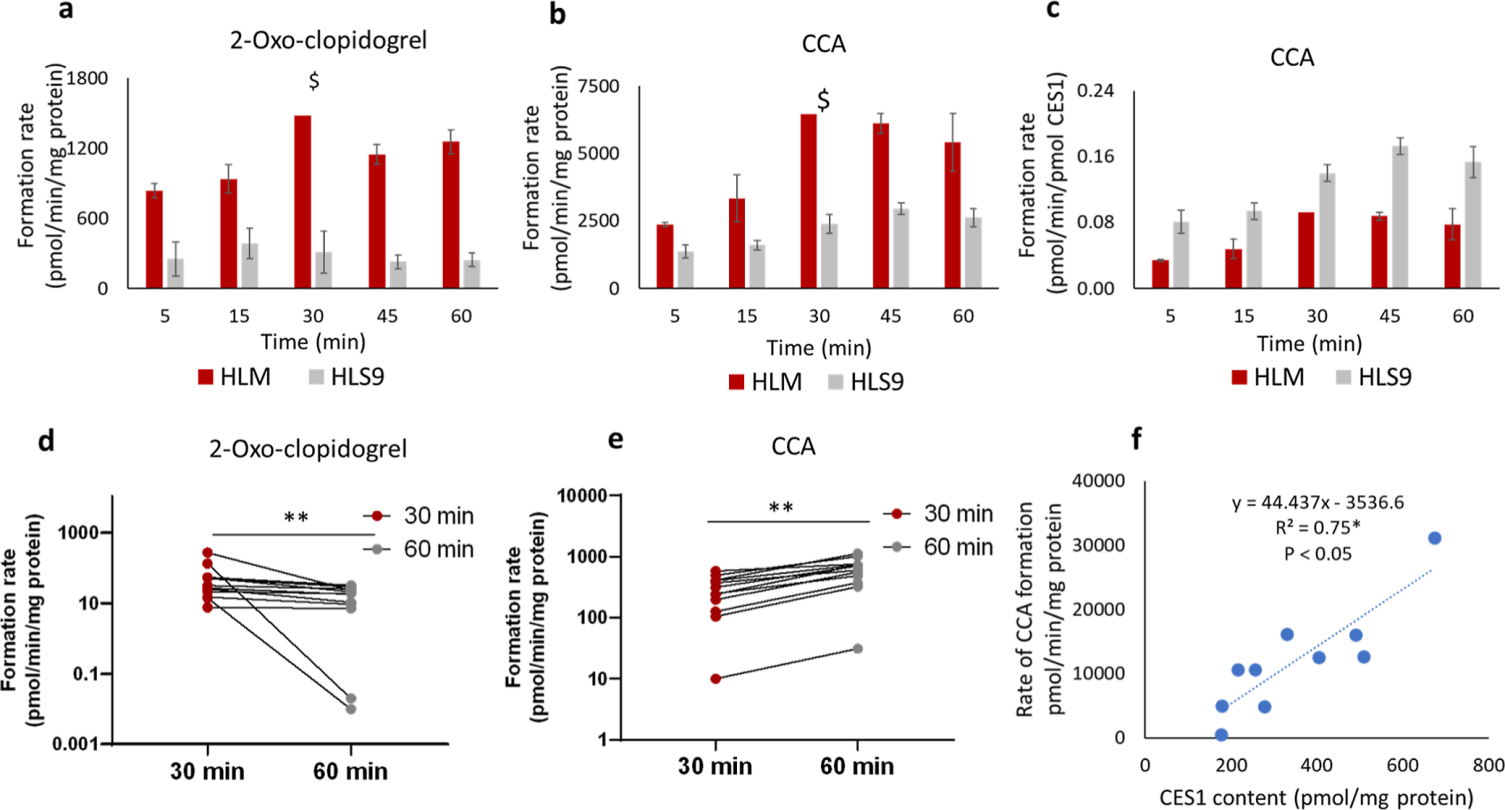
Time-dependent formation of 2-oxo-clopidogrel (a) and CCA (b) and CES1 normalized formation of CCA (c) in pooled HLM and pooled HLS9 samples (*n* = 3). The dollar symbol ($) represents duplicate samples. Comparison of rates of 2-oxo-clopidogrel (d) and CCA (e) formation in individual HLMs at 30- and 60 min incubation time, **p < 0.005, Wilcoxon test. Correlation of CES1 content with the rate of CCA formation in individual HLM samples (f).

The rate of CCA formation varied around 50-fold, while that of 2-oxo clopidogrel varied by 36-fold between the donors with highest and lowest activities among individual donors at the 30 min incubation time (Figure 4a). A moderate correlation (*R* ^2^ = 0.35 and *P* = 0.07) was observed between the ratios of CES1/CYP3A4 abundance to the ratios of CCA/2-oxo-clopidogrel formation rates (Figure 4b). The formation of CCA and 2-oxo-clopidogrel was higher in HLM5 and HLM10, respectively, reflecting the corresponding ratios of CES1 to CYP3A4 abundance in these samples (Figure 4c). A poor correlation (*r*2 = 0.0858 and *p* = 0.4) was observed between the ratios of CES1/CYP2C19 abundance to the ratios of CCA/2-oxo-clopidogrel formation rates (Figure 4d). The formation of CCA and 2-oxo-clopidogrel in HLM5 and HLM10 did not align with the corresponding ratios of CES1 to CYP2C19 abundance in these samples (Figure 4e). The ratios of protein abundances of CES1 and CYP3A4 and CES1 and CYP2C19 in all individual HLM samples (*n* = 10) (Supporting Information Figure S3a,b).

**Figure 4:**
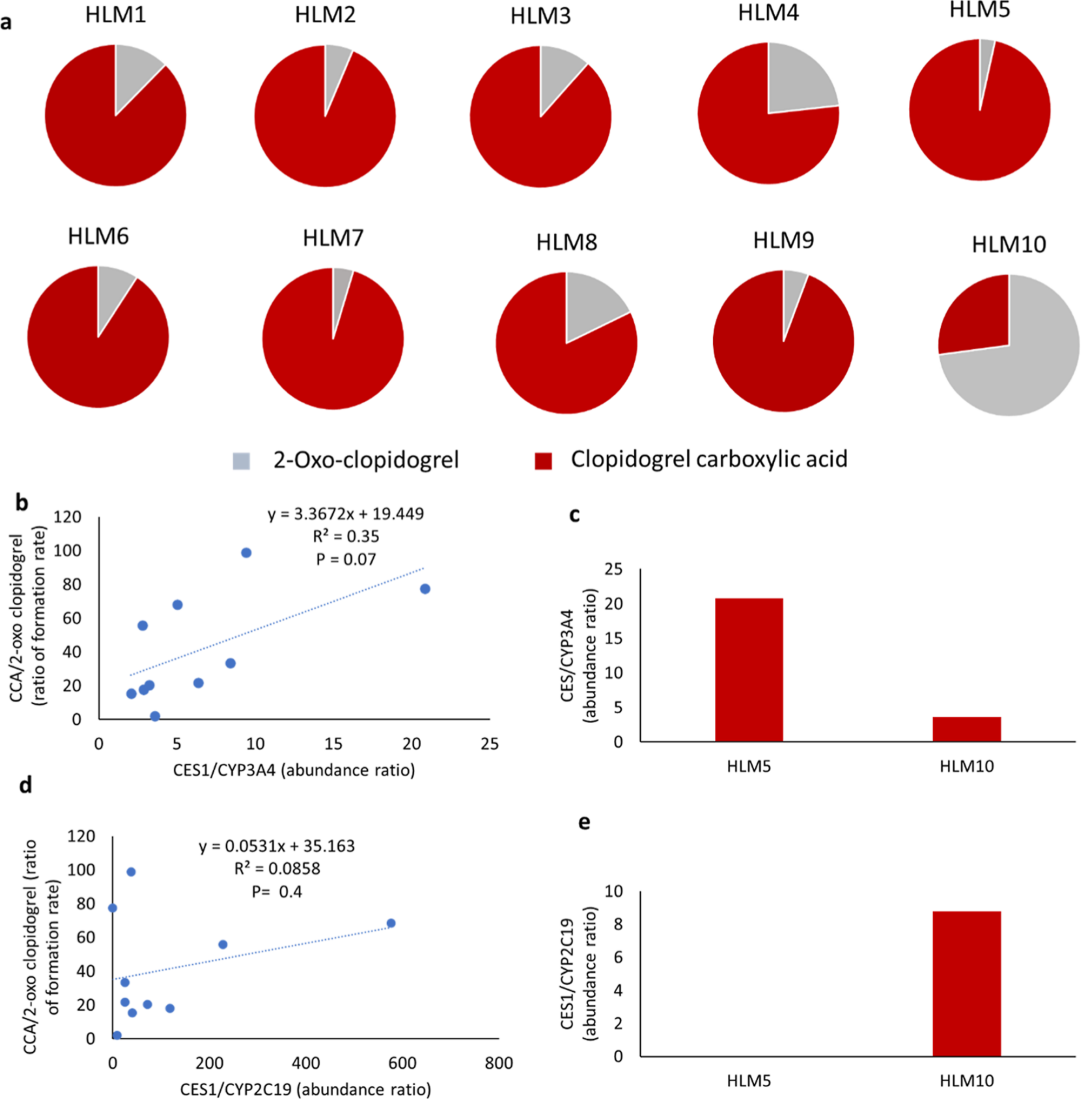
Relative formation of 2-oxo-clopidogrel and clopidogrel carboxylic acid in individual HLMs (*n* = 10) at 30 min time point. (a) Correlation of CES1/CYP3A4 abundance ratio to CCA/2-oxo-clopidogrel formation ratio in individual HLM samples (*n* = 10), (b) ratio of protein abundances of CES1 and CYP3A4 in HLM5 and HLM10, (c) correlation of CES1/CYP2C19 abundance ratio to CCA/2-oxo-clopidogrel formation ratio in individual HLM samples (*n* = 10), and (d) ratio of protein abundances of CES1 and CYP2C19 in HLM5 and HLM10 (e).

The formation of both 2-oxo-clopidogrel and CCA exceeded the linear range at 0.5 mg/mL protein concentration. Thus, protein concentrations of 0.2 and 0.1 mg/mL, with incubation times of 5 and 30 min, were used to determine the enzyme kinetics of 2-oxo-clopidogrel and CCA, respectively.

### 3.3 Relative Content of CES1, CYP3A4, and CYP2C19 and Formation of 2-Oxo-clopidogrel and CCA in Hepatocytes from Individual Donors

The relative contents of CES1, CYP3A4, and CYP2C19 (pmol/mg protein), along with the formation rates of 2-oxo-clopidogrel and CCA in hepatocytes from three individual donors (HH1, HH2, and HH3), are shown in Figure 5. CES1 abundance aligned with the CCA formation, which was highest in HH1, followed by HH2 and HH3. Whereas CYP3A4 and CYP2C19 abundances were highest in HH1, with comparable levels observed in HH2 and HH3, 2-oxo-clopidogrel formation was highest in HH3. These data suggest the confounding effect of CES1 abundance on oxidative metabolism.

**Figure 5:**
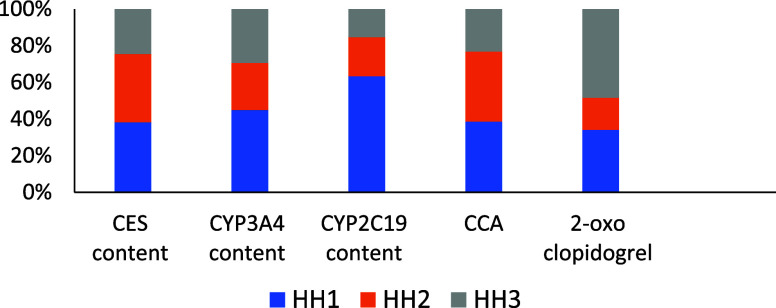
Relative content of CES1, CYP3A4, and CYP2C19 (pmol/mg) and relative formation of CCA and 2-oxo-clopidogrel in individual hepatocytes (HH1, HH2, and HH3).

### 3.4 Kinetics of 2-Oxo-clopidogrel and CCA Formation in the Recombinant Human Enzymes

Michaelis–Menten kinetics were applied to the formation rates of 2-oxo-clopidogrel and CCA in rCYPs 1A2, 2B6, 2C9, 2C19, 3A4, and rCES1 (Figure 6). The *K*_m_ and *V*_max_ values for CCA formation in rCES1 were 14.92 ± 4.8 μM and 2353 ± 144 pmol/min/mg protein, respectively.

**Figure 6:**
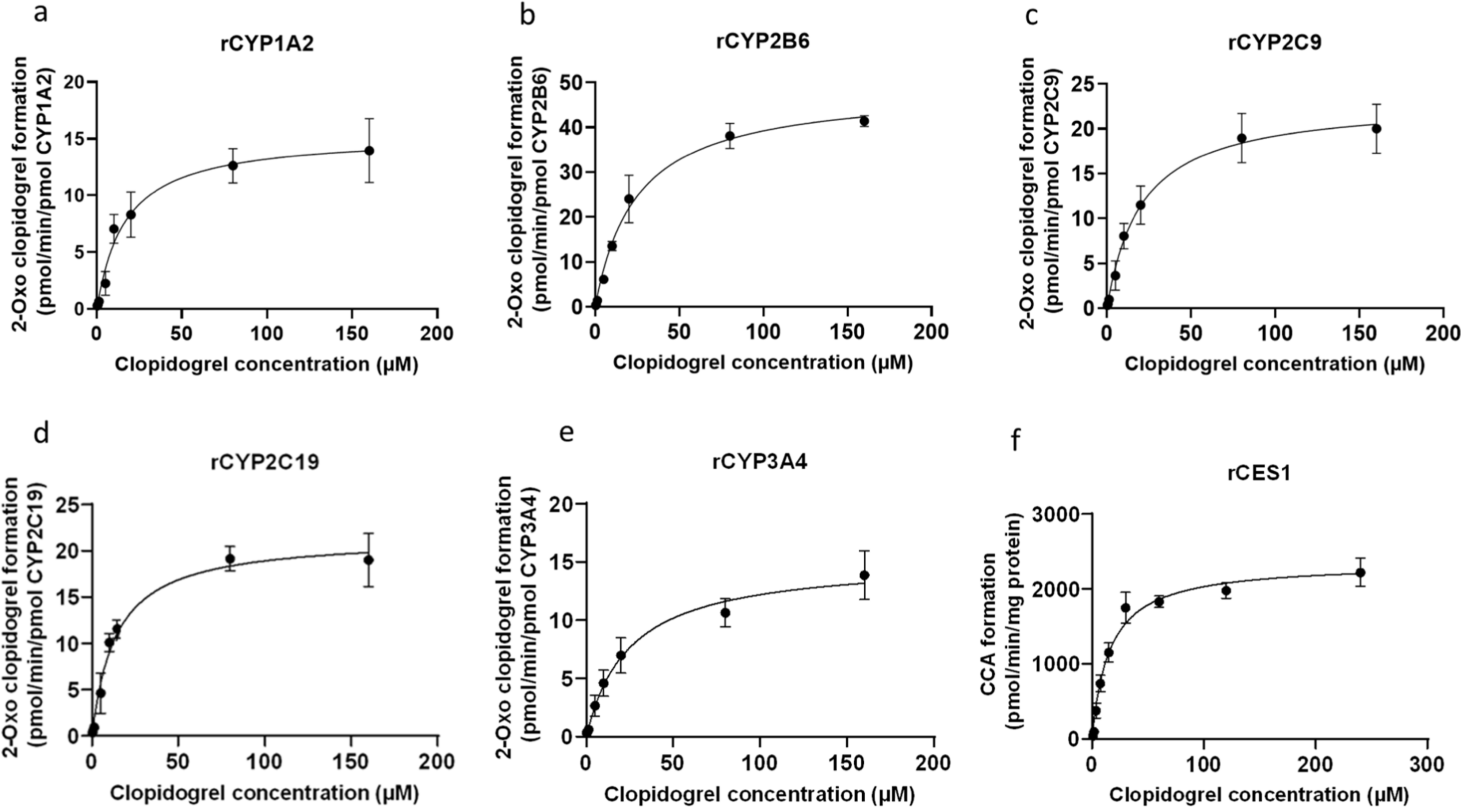
Kinetics of formation of 2-oxo-clopidogrel formation in rCYP1A2 (a), rCYP2B6 (b), rCYP2C9 (c), rCYP2C19 (d), rCYP3A4 (e), and of clopidogrel carboxylic acid formation in rCES1 (f).

The *K*_m_ values for 2-oxo-clopidogrel formation ranged from 13.8 ± 5.5 μM for rCYP2C19 to 34.5 ± 9.5 μM for rCYP2B6 (Table 2). The CL_int_ values (μL/mg protein/min) were determined by scaling *V*_max_/*K*_m_ with the respective abundances of CYP isoforms in pooled HLM.

**Table 2:** Kinetic Parameters of Formation of 2-Oxo-clopidogrel Formation and % Fraction Metabolized (*f*_m_) by CYP1A2, CYP2B6, CYP2C9, CYP2C19, and CYP3A4

CYP isoform	*V*_max_ (pmol/min/pmol P450)	*K*_m_ (μM)	CL_int_ (*V*_max_/*K*_m_) (μL/pmol P450/min)	Abundance in pooled HLM (pmol/mg protein)	CL_int_ (μL/mg protein/min)	REF	CL_int_ (μL/mg protein/min) *REF	*f*_m_ (%)
CYP1A2	15.5	17.0	0.9	14.1	12.7	0.0028	0.04	0.6
CYP2B6	60.8	34.5	1.8	8.9	15.8	0.0054	0.09	1.5
CYP2C19	21.6	13.8	1.6	2.8	4.4	0.0018	0.01	0.1
CYP2C9	23.2	20.6	1.1	87.5	98.3	0.0214	2.11	40.3
CYP3A4	15.2	24.7	0.6	137.4	84.5	0.0353	2.99	57.2

The highest CL_int_ value for 2-oxo-clopidogrel formation (μL/mg protein/min) was 98.3 (CYP2C9), followed by 84.5 (CYP3A4), while the lowest CL_int_ value was 4.4 (CYP2C19). Following correction with their respective REF, the highest contribution toward 2-oxo-clopidogrel formation was CYP3A4 and CYP2C9, with *f*_m_ values of 57.2% and 40.3% respectively.

### 3.5 Inhibition of 2-Oxo-clopidogrel Formation Using Sulfaphenazole and CYP3cide in Pooled HLM

The formation of 2-oxo-clopidogrel was strongly inhibited in pooled HLM in the presence of the CYP3A4-specific inhibitor, CYP3cide (40.8%), whereas the CYP2C9-specific inhibitor, sulfaphenazole, showed an inhibition of only 11% (Supporting Information Figure S4).

## 4 Discussion

Clopidogrel is commonly prescribed as a first-line antiplatelet agent due to its efficacy and cost-effectiveness.^28^ Despite its widespread use, significant interindividual variability in clopidogrel response has been observed, leading to inconsistent therapeutic outcomes among patients. In response, the U.S. food and drug administration (FDA) issued a black box warning for clopidogrel, alerting that certain patients do not effectively activate the drug, thereby receiving reduced therapeutic benefits.^29^

Clopidogrel metabolism involves both CES1 and CYP enzymes, and therefore, the *f*_m_ of these enzymes should be accurately estimated to better understand their impact in the variability in the therapeutic response.

The choice of appropriate in vitro models is crucial for characterizing metabolism by different enzyme classes and accurately translating in vitro data into the in vivo context. While drug metabolizing CYP enzymes are well-characterized as being located on the membrane of the endoplasmic reticulum (ER), the localization of CES1 has been ambiguous until recently. Initially thought to be on the ER membrane,^30^ CES1 was later clarified to be localized in the ER lumen.^31^ The preparation of subcellular fractions, such as HLS9 and HLM, involves homogenization and differential centrifugation. During the preparation of S9, the ER luminal content, including proteins like calreticulin and CES1, is released into the cytosolic matrix. In contrast, during the preparation of microsomes, vesicles are formed from the ER, resulting in CES1 being encapsulated by the ER membrane. This is evident from activity assays in the subcellular fractions, which revealed a higher specific rate of CCA formation in the S9 fraction compared to HLM. These findings support the greater substrate accessibility to CES1 in S9 fractions. This observation, coupled with the strong correlation with the ER lumen marker, calreticulin, confirms CES1 localization in the lumen. The differential localization of CYP versus CES1 enzymes is a critical factor that is not currently considered into in vitro to in vivo extrapolation models, potentially leading to inaccuracies in estimating the fractional contributions of these enzymes. Furthermore, variability in the ratio of ER markers (calreticulin to calnexin) in individual HLM samples suggests technical variability in HLM preparation, indicating that HLM may not be a suitable in vitro model for dual substrates metabolized by both CES1 and CYP enzymes.

Clinically, CYP2C19 has been established as a key enzyme influencing the formation of the active metabolite of clopidogrel and its associated therapeutic outcomes.^32^ Approximately 85% of clopidogrel dose is inactivated by CES1; thus, any changes in CES levels or activity could significantly influence the levels of the active clopidogrel metabolite and consequently its efficacy. However, compared to CYP enzymes, the impact of CES1 variability on the PK and PD of clopidogrel remains understudied.^28^ CES1 expression and activity vary widely among individuals due to genetic and nongenetic factors contributing to significant PK/PD variability. A study evaluating 20 CES1 SNPs reported substrate-dependent effects ranging from a complete loss to a moderate reduction in activity. Many of these variants occur at appreciable allele frequencies (≥2%) across populations, highlighting potential clinical relevance.^33^ In addition to genetic polymorphisms, CES1 activity is modulated by epigenetic changes, age, sex, liver disease, inhibitors, and inducers, though their molecular mechanisms remain unclear. In addition to age-dependent changes, a proteomic analysis of liver microsomes from 35 adults revealed a 158-fold variation in CES1 expression, indicating substantial interindividual variability in drug metabolism.^34^ Despite extensive in vitro and animal data on CES1 modulators, few DDIs are confirmed clinically, and no CES1 pharmacogenomic biomarkers are incorporated into CPIC guidelines or FDA labeling.^35^ A few studies have investigated the effect of the CES1 G143E variant on clopidogrel PK and PD profiles. Carriers of the G143E allele exhibited 50% higher concentrations of the active metabolite and 24% greater platelet aggregation inhibition relative to noncarriers.^4,36,37^ Similarly, it was found that the variant G143E carriers had a 53% lower AUC ratio of CCA to clopidogrel and higher plasma concentrations of clopidogrel and its active metabolite, accompanied by 19% increase in platelet aggregation inhibition compared to noncarriers.^22,38^ These findings highlight the significant role that CES1 protein variability plays in the metabolic activation of clopidogrel.

Our proteomics data revealed wide variability in CES1 content across HLM samples from individual donors, correlating with the relative formation of CCA versus 2-oxo clopidogrel in these samples. Specifically, CCA formation in hepatocytes aligned with CES1 content, whereas 2-oxo-clopidogrel formation did not align with CYP3A4 and CYP2C19 levels. Interestingly, the highest 2-oxo-clopidogrel formation was observed in the donor with the lowest CES content, likely due to an increased availability of the parent compound and reduced formation of CCA. This observation emphasizes the critical role of CES1 levels in modulating 2-oxo-clopidogrel formation and potentially affecting the formation of the downstream active metabolite.

Previous studies assessing CYP isoform contributions to clopidogrel metabolism have employed various approaches including substrate depletion, correlation analyses, CES inhibition, and recombinant enzyme studies.^2,8,39–41^ For example, it was previously reported that CYP2C19 and CYP1A2 had the highest *f*_m_ (%) toward 2-oxo-clopidogrel formation, followed by CYP2B6, while CYP2C9 and CYP3A4 were not involved in metabolite formation.^8^ In contrast, our findings indicate that both CYP3A4 and CYP2C9 contribute to 2-oxo-clopidogrel formation, as evidenced by inhibition in the presence of sulfaphenazole and CYP3cide. The CYP2C9 inhibition experiment aimed to qualitatively demonstrate that clopidogrel metabolism can be inhibited by a CYP2C9 inhibitor. As a low concentration of sulfaphenazole was used, complete inhibition was unlikely, resulting in a lower *f*_m_ than that obtained using the REF approach. Due to potential cross-reactivity, inhibition assays for other CYP isoforms were not conducted. Clinical data further support the role of CYP3A4 in modulating the PK of the active clopidogrel metabolite and its PD in subjects receiving the drug alone or coadministered with the CYP3A inhibitors ketoconazole and itraconazole.^42,43^ In the presence of these inhibitors, a reduction in both the exposure and the concentration of the active metabolite was observed, leading to a corresponding decrease in platelet aggregation inhibition. Ketoconazole reduced the AUC_0–24_ by 22% after a 300 mg loading dose (LD) and by 29% after a 75 mg maintenance dose (MD). Additionally, the *C*_max_ of the active metabolite decreased significantly by 48% and 61% following LD and MD, respectively. Conversely, an increase in active metabolite formation (∼four-fold increase in AUC) was seen with rifampicin (CYP3A4 inducer), leading to an increase in platelet aggregation.^44^ The loss of function variants of CYP2C9 were significantly associated with lower exposure (*P* = 0.043), lower *C*_max_ (*P* = 0.006), and poor responder status (*P* = 0.006) in subjects receiving clopidogrel.^16^ However, these studies do not clarify whether CYP3A4 and CYP2C9 contribute to the formation of the 2-oxo-clopidogrel intermediate, the active metabolite, or both. In our study, the formation of the active metabolite was not directly monitored due to its high reactivity and challenges in assay development, representing a limitation. However, the primary objective of our study was to demonstrate how interindividual variability in CES1-mediated metabolism can confound the interpretation of CES and CYP contributions. To address this, we focused on the relative formation of CCA and 2-oxo-clopidogrel, which could directly influence active metabolite levels. This approach enabled us to evaluate the interplay between CES1 and CYP pathways within the study’s scope.

Our data does not align with greater importance of CYP2C19 in 2-oxo formation.^8^ This discrepancy can likely be explained by (1) the difference in recombinant systems used (Supersomes by Kazui et al. versus Bactosomes in our study). (2) Furthermore, Kazui et al. did not detect the 2-oxo metabolite in rCYP2C9 and rCYP3A4 incubations, possibly due to limited activity of the rCYPs employed.^8^ CYP2C19 is well established in the formation of clopidogrel’s active metabolite, but its role in forming the primary oxidative metabolite, 2-oxo-clopidogrel, is debated. Previous studies assessed CYP3A4 and CYP2C19 using recombinant enzymes and inhibitors of other CYPs to estimate isoform contributions.^45^ However, inhibitor selectivity is limited, and off-target effects may confound results—for instance, CYP3A4 inhibitors reduced clopidogrel metabolism by 50%–70%, while the CYP2C19 inhibitor omeprazole caused only a 15%–20% decrease, which could reflect nonspecific inhibition. Our approach offers greater selectivity, providing a clearer estimate of the relative contributions (*f*_m_) of individual CYP enzymes. Nevertheless, our finding of low contribution of CYP2C19 toward 2-oxo clopidogrel formation warrants further investigation. It is also important to note that the association of CYP2C19 polymorphisms with clopidogrel efficacy is primarily driven by the active metabolite formation from 2-oxo clopidogrel.

In conclusion, our study highlights the need to understand the confounding effects of CES1 and CYP enzymes in the oxidation of dual substrates, using clopidogrel as an example. Our findings suggest that HLM may not be an ideal in vitro model for *f*_m_ estimation for dual substrates due to the differential subcellular localization of CES and CYP enzymes. Hepatocytes, with their intact subcellular architecture, may provide a more accurate model for these estimations. Our study identified CYP3A4 as the primary enzyme responsible for clopidogrel oxidation to 2-oxo-clopidogrel with CYP2C9 playing a secondary role, which is a novel finding. In addition, the substantial role of CES1 in modulating interindividual variability in clopidogrel disposition has often been overlooked. Given that 85% of clopidogrel is inactivated by CES1, genetic variations in CES1 or coadministration with CES1 inhibitors could significantly affect the formation of the 2-oxo metabolite and consequently the active metabolite. We propose that physiologically based pharmacokinetic models, incorporating variability in both CYP and CES1 enzymes, should be considered for *f*_m_ estimation for dual substrates. Additionally, genotyping-guided or biomarker-informed approaches should account for the variability in both CYP and CES1 enzymes to optimize clopidogrel therapy.

## Supplementary Material



## Notes

MS method for the analysis of clopidogrel metabolites on HRMS; relative abundance of CYP1A2, CYP2B6, CYP2C9, CYP2C19, and CYP3A4 in individual HLM, pooled HLM, pooled HLS9, and hepatocyte samples from 3 individual donors; comparison of replicates in TPA-based global proteomics analysis of CYP isoforms and CES1 individual HLM (*n* = 10), pooled HLM (*n* = 150), pooled HLS9 (*n* = 50), and individual hepatocytes (*n* = 3); correlation of CES1 with calnexin, calreticulin, **p* < 0.05, and degree of physiological resemblance of individual HLMs to ER; ratio of protein abundances of CES1 and CYP3A4 and CES1 and CYP2C19 in individual HLM samples (*n* = 10); and inhibition of 2-oxo-clopidogrel formation using sulfaphenazole and CYP3cide (PDF)

## ¶

Cincinnati Children’s Hospital Medical Center, Cincinnati, Ohio, 45229, United States

## ∇

Drug Metabolism and Pharmacokinetics, Treeline BioSciences, San Diego, California 92121, United States.

## Abbreviations Used

CYP,: cytochrome P450
CES: carboxylesterase
HLM: human liver microsomes
HLS9: human liver S9
rCYP: recombinant cytochrome P450 enzyme
rCES: recombinant carboxylesterase enzyme
*f*_m_: fraction metabolized
LC-MS/MS: liquid chromatography tandem mass spectrometry
PK: pharmacokinetics
PD: pharmacodynamics
HRMS: high-resolution mass spectrometry
