# Supplementary material for: Confounding Effect of Hepatic Carboxylesterase 1 (CES1) Variability on Clopidogrel Oxidation
Source: Mol Pharm. 2025 Nov 13;22(12):7359–70. doi: 10.1021/acs.molpharmaceut.5c00462 (PMC12673578; doi:10.1021/acs.molpharmaceut.5c00462)
Supplement: Supplementary file 1 [file mp5c00462_si_001.pdf]

Title: The confounding effect of hepatic carboxylesterase 1 (CES1) variability on clopidogrel oxidation

Authors:

Sandhya Subash[1,2], Dilip K. Singh[1,2], S. Cyrus Khojasteh[3], Bernard P. Murray[4], Michael A. Zientek[5,6], Robert S. Jones[3], Priyanka Kulkarni[7], Bill J. Smith[8], Bhagwat Prasad[1,2]

1. College of Pharmacy and Pharmaceutical Sciences, Washington State University (WSU), Spokane, WA 99202, United States
2. Cincinnati Children's Hospital Medical Center, Cincinnati, OH (current affiliation), 45229, United States
3. Drug Metabolism and Pharmacokinetics, Genentech, Inc., South San Francisco, CA, 94080, United States
4. Drug Metabolism, Gilead Sciences, Inc., Foster City, CA, 94404, United States
5. Drug Metabolism and Pharmacokinetics, Takeda Development Center Americas, Inc., San Diego, CA, 92121, United States
6. Drug Metabolism and Pharmacokinetics, Treeline BioSciences, San Diego, CA, 92121, United States (current affiliation)
7. Drug Metabolism and Pharmacokinetics Takeda Pharmaceuticals, Inc., Cambridge, MA, 02139, United States
8. Terminal Phase Consulting LLC, Colorado Springs, CO, 94404, United States

Supplementary Tables:

**Table S1:** MS conditions used for the analysis of clopidogrel metabolites on HRMS		
Full MS	Run Time	0 to 30 min
	Polarity	Positive
	Insource CID	0.0 eV
	Default charge	1
	Spectrum data type	Profile
Tandem mass (MS/MS)	Microscans	1
	Resolution	30,000
	AGC target	2.00E+05
	Maximum IT	100 ms
	Loop count	6
	MSX count	1
	Isolation window	1.5 m/z
	Isolation offset	0.0 m/z
	Normalized CE	19
	Spectrum data type	Centroid

Table S2: Relative abundance of CYP1A2, CYP2B6, CYP2C9, CYP2C19, and CYP3A4 in individual HLM, pooled HLM, pooled HLS9 and hepatocyte samples from 3 individual donors

Matrix	Protein abundance (pmol/mg protein)					
	CYP1A2	CYP2B6	CYP2C9	CYP2C19	CYP3A4	CES1
HLM_1	131.53	8.42	90.74	19.91	77.15	490.59
HLM_2	22.34	4.58	73.91	10.68	43.04	404.75
HLM_3	17.13	5.97	54.14	0.88	101.10	508.35
HLM_4	29.96	9.89	76.86	2.34	96.71	277.63
HLM_5	19.64	1.79	66.50	0.00	15.87	330.64
HLM_6	14.01	89.95	53.49	2.95	241.91	673.22
HLM_7	32.53	72.60	56.65	2.98	67.00	215.08
HLM_8	57.13	44.07	81.39	6.31	124.00	255.43
HLM_9	22.34	2.29	65.55	7.21	21.27	178.34
HLM_10	45.41	4.95	79.18	20.10	49.29	176.54
Pooled HLM	14.05	8.98	87.52	2.83	137.36	470.53
Pooled HLS9	6.83	4.15	20.04	1.49	22.86	100.50

**HH1**	**1.76**	**0.65**	**13.67**	**0.94**	**8.49**	**111.06**
**HH2**	**2.62**	**0.00**	**6.19**	**0.32**	**4.86**	**108.05**
**HH3**	**2.44**	**0.44**	**11.12**	**0.23**	**5.57**	**71.25**

Supplementary Figures

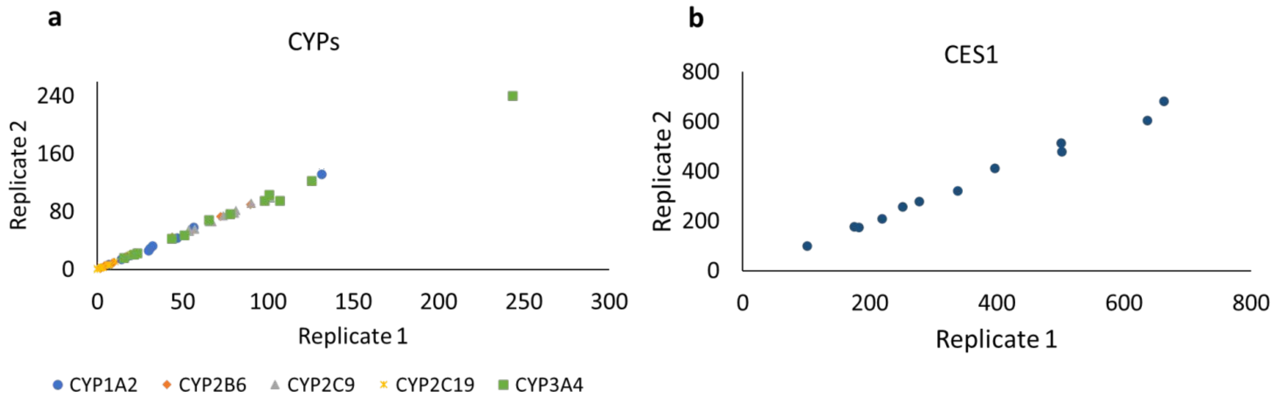

Fig S1: Comparison of replicates in TPA-based global proteomics analysis of CYP isoforms (a) and CES1 (b) individual HLM (n=10), pooled HLM (n=150), pooled HLS9 (n=50).

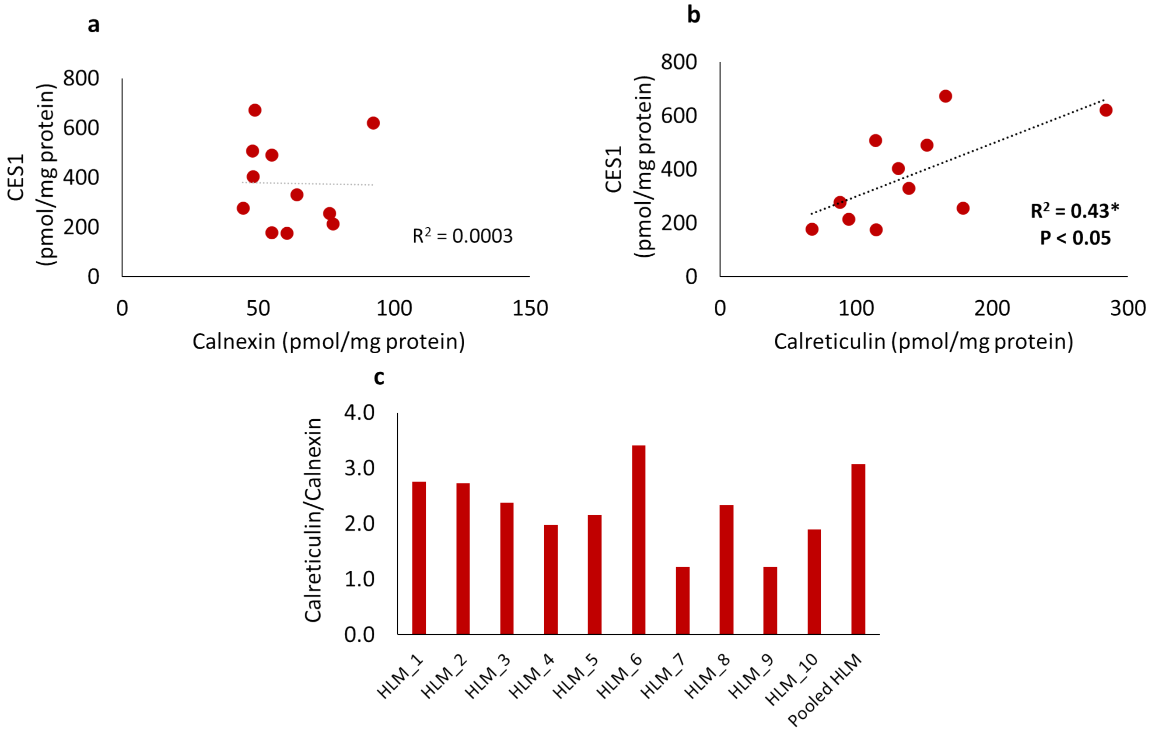

Fig. S2: Correlation of CES1 with calnexin (a), calreticulin(b) *p <0.05, and degree of physiological resemblance of individual HLMs to ER (c).

a

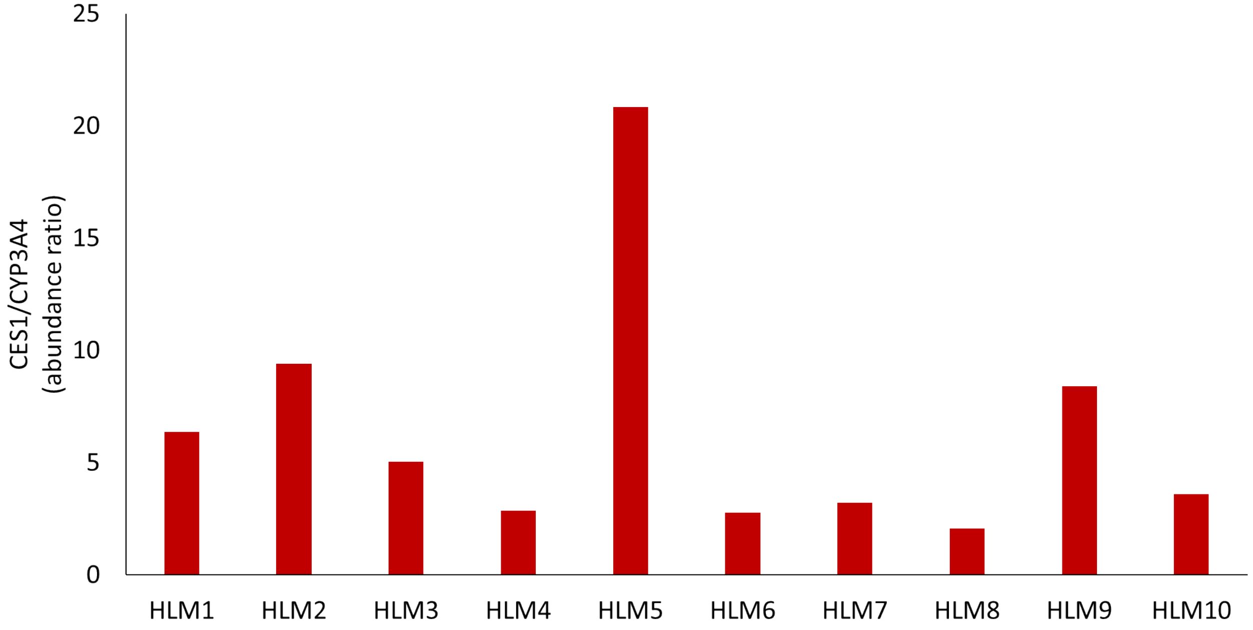

b

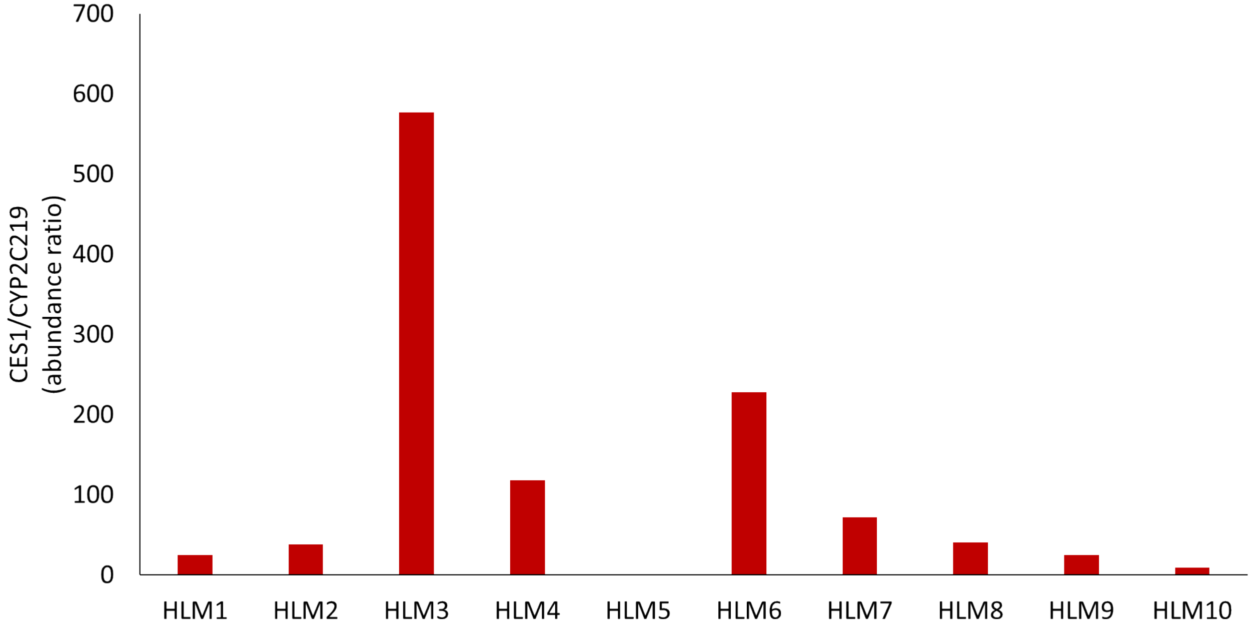

Fig. S3: Ratio of protein abundances of CES1 and CYP3A4 (a) and CES1 and CYP2C19 (b) in individual HLM samples (n=10)

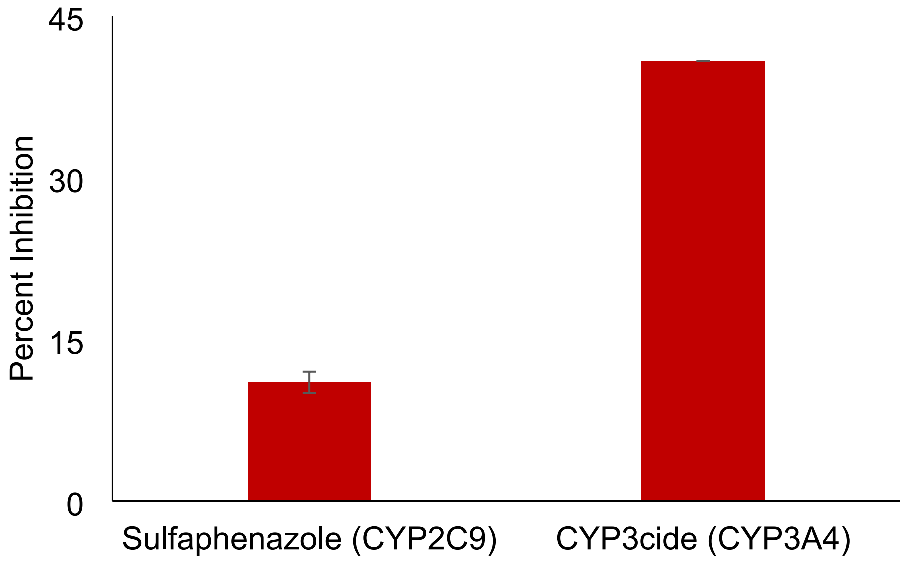

Fig. S4: Inhibition of 2-oxo-clopidogrel formation using sulfaphenazole and CYP3cide
